# Transformative potential of Artificial Intelligence in pharmacy practice

**DOI:** 10.1016/j.jsps.2023.101706

**Published:** 2023-07-22

**Authors:** Muhammad Ahmer Raza, Shireen Aziz

**Affiliations:** Department of Social and Clinical Pharmacy, Faculty of Pharmacy in Hradec Králové, Charles University in Prague, Czech Republic; Faculty of Pharmacy, The University of Faisalabad, Faisalabad, Pakistan; Department of Social and Clinical Pharmacy, Faculty of Pharmacy in Hradec Králové, Charles University in Prague, Czech Republic; Faculty of Pharmacy, The University of Faisalabad, Faisalabad, Pakistan

*Dear Editor*

We are writing to express our deep appreciation for the article in *Saudi Pharmaceutical Journal*, available online July 7, 2023, titled ‘Artificial intelligence in pharmacy practice: Attitude and willingness of the community Pharmacists and the barriers for its implementation’ by Anan S. Jarab et al., to highlight the advancement in technology at an unprecedented pace and Artificial Intelligence (AI) holds great promise in revolutionizing the way pharmacists can deliver patient care, optimize medication management, and improve overall health outcomes. In addition, we will provide recommendations for how successfully we can integration AI in pharmacy practice.

AI has emerged as a transformative force across various industries, revolutionizing the way we work, communicate, and live. In the field of healthcare, AI holds immense potential to improve patient outcomes, enhance efficiency, and drive innovation. With its ability to analyze vast amounts of data, recognize patterns, and make intelligent predictions, AI has the potential to address key challenges in healthcare and reshape the future of medicine. AI algorithms have shown remarkable capabilities in analyzing medical images and assisting in diagnostics. Deep learning algorithms can analyze radiology images, pathology slides, and dermatological images with high accuracy. This enables early detection of diseases, more accurate diagnoses, and improved treatment planning. AI-powered diagnostics can help healthcare professionals make faster and more informed decisions, leading to better patient outcomes (Yu et al., 2018).

Pharmacy practice, as an integral part of the healthcare system, can greatly benefit from the adoption of AI technologies. Pharmacy practice plays a vital role in healthcare, encompassing medication management, patient counseling, and medication safety (Mohiuddin, 2020). However, the traditional practice of pharmacy faces numerous challenges, including medication errors, drug interactions, and suboptimal medication adherence. These challenges can have significant consequences for patient health and well-being. AI technologies offer a promising solution to address these challenges and revolutionize pharmacy practice. AI-powered systems can significantly assist in these areas, leveraging advanced algorithms and machine learning techniques to analyze vast amounts of patient data and provide tailored medication regimens. By integrating patient-specific information, such as medical history, genetic profiles, and drug interactions, AI algorithms can generate personalized medication recommendations. This not only reduces the risk of adverse drug events but also enhances treatment effectiveness, particularly in complex patient cases. AI can also automate medication reconciliation and drug interaction checks, providing real-time alerts and warnings to pharmacists, improving medication safety and reducing errors (Raza et al., 2022).

AI has the potential to revolutionize the drug discovery and development process, which is traditionally time-consuming, expensive, and prone to failure. AI algorithms can analyze large datasets and predict the efficacy and safety of potential drug candidates. By simulating molecular interactions and predicting drug-target interactions, AI can assist researchers in prioritizing and optimizing compounds, significantly reducing the time and resources required for preclinical and clinical trials. The integration of AI technologies in pharmacy practice enables the use of predictive analytics for early detection and intervention. By analyzing electronic health records, genetic information, and other relevant data, AI algorithms can help healthcare professionals predict diseases, identify at-risk populations, and personalize treatment plans. Early detection leads to timely interventions, improving patient outcomes and reducing healthcare costs (Raza et al., 2022).

AI-powered decision support systems can provide real-time guidance to pharmacists, incorporating evidence-based guidelines, clinical databases, and patient-specific data (Mishra, 2018). These systems assist in assessing drug interactions, recommending appropriate therapies, and identifying potential adverse effects. By augmenting human expertise, AI enhances the quality and consistency of decision-making, reducing the risk of errors and improving patient safety. In addition to improving healthcare delivery, AI technologies can also enhance patient engagement and education. Chatbots, virtual assistants, and mobile applications equipped with natural language processing capabilities can provide personalized education, medication reminders, and adherence monitoring. Patients can interact with these AI-driven systems to receive information about their medications, ask questions, and access resources (Ramadhani, 2023). These interventions promote medication adherence, improve patient satisfaction, and alleviate the burden on healthcare providers. Compare the advantages and limitations of AI integration in pharmacy practice with traditional methods we can gain insights into the transformative potential of AI and understand how it enhances the traditional practice of pharmacy. Table 1 provides the summary of comparison of AI integration in pharmacy practice with traditional methods.

**Table 1:** Comparison of AI integration in pharmacy practice with traditional methods.

Areas of comparison	Traditional Method	AI Integration
Medication management and optimization	Manual Prone to error Limited processing capability	Accurate and efficient Reduce risk of error Personalized medication
Drug discovery and development	Time consuming and expensive Trial error and methods Limited success rates in drug development	Accelerate drug discovery and development Predict drug safety and efficacy Increase success rate in drug development
Clinical decision support	Rely on human expertise Time consuming and subject to human error Limited data consideration	Real time access to evidence-based guidelines Improve accuracy and consistency in decision making Enhance patient safety and outcomes
Predictive Analytics and Early Intervention	Rely on retrospective analysis Limited data processing capabilities Reactive healthcare management	Early detection and intervention of disease Personalized treatment plans based on big dataset Proactive preventive strategies

To ensure the successful integration of AI in pharmacy practice, several recommendations should be considered. Firstly, there is a need for robust data collection and sharing systems that allow pharmacy institutions to aggregate and anonymize the curriculum. Collaborative efforts between academic institutions, healthcare providers, and industry stakeholders are crucial for establishing comprehensive databases for pharmacy practice that can train pharmacist. Secondly, AI algorithms need to be continuously validated and updated using real-world data. Pharmacy is a dynamic field, and the performance of AI models must be regularly assessed to ensure accuracy and reliability. Ongoing feedback loops between AI systems and pharmacy institutions are essential for refining and optimizing these models over time. Lastly, pharmacists should be provided with adequate training and education on the utilization of AI tools in practice. Understanding the strengths and limitations of AI systems will enable pharmacists to interpret AI-generated insights effectively and make informed decisions for their patients.

To conclude, AI has the potential to revolutionize pharmacy field worldwide, transforming medication management, drug discovery, and patient care. By leveraging AI technologies, pharmacists can enhance medication safety, optimize treatment outcomes, and improve patient engagement. In addition, the integration of AI in pharmacy practice will further offer numerous advantages over traditional methods. AI enhances medication management and optimization by providing personalized regimens and reducing the risk of errors. In drug discovery and development, AI accelerates the process and increases success rates. Clinical decision support with AI improves accuracy and consistency. AI-powered predictive analytics enable early intervention, personalized treatment plans, and targeted preventive strategies. Furthermore, AI technologies continue to evolve, it is essential for pharmacists to stay updated with the latest advancements. Engage in continuous professional development and seek opportunities to learn about AI applications in pharmacy practice. Stay informed about new tools, algorithms, and guidelines that can enhance your practice and improve patient care and develop a solid understanding of AI algorithms commonly used in pharmacy practice. Familiarize yourself with their strengths, limitations, and potential biases. This knowledge will enable pharmacists to critically evaluate AI-generated insights and make informed decisions in collaboration with AI technologies.

## Funding

None.

## Declaration of Competing Interest

The authors declare that they have no known competing financial interests or personal relationships that could have appeared to influence the work reported in this paper.
